# Chronic Administration of Proanthocyanidins or Docosahexaenoic Acid Reversess the Increase of miR-33a and miR-122 in Dyslipidemic Obese Rats

**DOI:** 10.1371/journal.pone.0069817

**Published:** 2013-07-26

**Authors:** Laura Baselga-Escudero, Anna Arola-Arnal, Aïda Pascual-Serrano, Aleix Ribas-Latre, Ester Casanova, M-Josepa Salvadó, Lluis Arola, Cinta Blade

**Affiliations:** Department of Biochemistry and Biotechnology, Universitat Rovira i Virgili, Tarragona, Spain; Max Delbrueck Center for Molecular Medicine, Germany

## Abstract

miR-33 and miR-122 are major regulators of lipid metabolism in the liver, and their deregulation has been linked to the development of metabolic diseases such as obesity and metabolic syndrome. However, the biological importance of these miRNAs has been defined using genetic models. The aim of this study was to evaluate whether the levels of miR-122 and miR-33a in rat liver correlate with lipemia in nutritional models. For this purpose, we analyzed the levels of miRNA-33a and miR-122 in the livers of dyslipidemic cafeteria diet-fed rats and of cafeteria diet-fed rats supplemented with proanthocyanidins and/or ω-3 PUFAs because these two dietary components are well-known to counteract dyslipidemia. The results showed that the dyslipidemia induced in rats that were fed a cafeteria diet resulted in the upregulation of miR-33a and miR-122 in the liver, whereas the presence of proanthocyanidins and/or ω-3 PUFAs counteracted the increase of these two miRNAs. However, srebp2, the host gene of miR-33a, was significantly repressed by ω-3 PUFAs but not by proanthocyanidins. Liver mRNA levels of the miR-122 and miR-33a target genes, fas and pparβ/δ, cpt1a and abca1, respectively, were consistent with the expression of these two miRNAs under each condition. Moreover, the miR-33a and abca1 levels were also analyzed in PBMCs. Interestingly, the miR-33a levels evaluated in PBMCs under each condition were similar to the liver levels but enhanced. This demonstrates that miR-33a is expressed in PBMCs and that these cells can be used as a non-invasive way to reflect the expression of this miRNA in the liver. These findings cast new light on the regulation of miR-33a and miR-122 in a dyslipidemic model of obese rats and the way these miRNAs are modulated by dietary components in the liver and in PBMCs.

## Introduction

In the last decade, mi(cro)RNAs have emerged as a novel class of non-coding RNAs that are 20–25 nucleotides long and that regulate the expression of specific target genes at the post-transcriptional level, mainly by binding to the 3′ untranslated region (3′UTR) of target mRNAs. This triggers mRNA cleavage or inhibits translation [1]–[3]. miRNAs are known to modulate more than 60% of human transcripts and thus play important regulatory roles in a variety of biological processes and are implicated in almost all metabolic pathways [4]. Moreover, there is much evidence that the deregulation of miRNAs is related to the development of chronic diseases [5]. Specifically, miR-33 and miR-122 are known as major regulators of lipid metabolism in the liver, and their deregulation may contribute to the development of metabolic diseases such as obesity and metabolic syndrome [5], [6]. miR-122 plays a critical role in liver homeostasis by regulating genes with key roles in the synthesis of triglycerides (TGs) and fatty acids (FAs), such as FA synthase (FAS) and sterol regulatory element-binding protein 1c (SREBP1c), as well as genes that regulate FA β-oxidation [7], [8]. Moreover, gene silencing of miR-122 in mice [9], [10], African green monkeys [11] and chimpanzees [12], using either antagomirs or antisense oligonucleotides, significantly decrease plasma cholesterol and TG levels. Additionally, miR-33 plays an important role in the regulation of cholesterol homeostasis in the liver, regulating the ATP-binding cassette transporters (ABC transporters) ABCA1 and ABCG1 in addition to its role in FA β-oxidation by targeting the carnitine palmitoyltransferase 1a (CPT1a) [13]. Interestingly, miR-33 has two isoforms:miR-33b, which is present in a subset of species, such as dogs, pigs, non-human primates and humans, but not in rodents and miR-33a, which is highly conserved from humans to Drosophila. miR-33b and miR-33a isoforms are found in introns in the sterol response element binding protein 1 (SREBF1) and 2 (SREBF2) genes, respectively [13]–[15]. SREBF1 and SREBF2 code for the transcription factors SREBP1 and SREBP2, which regulate all SREBP-responsive genes in both the cholesterol and FA biosynthetic pathways [16]. Furthermore, the silencing of miR-33 by knockout or antisense techniques in mice results in an improvement in the plasma lipid profile, increasing plasma high density lipoprotein-cholesterol (HDL-C) levels [15], [17], [18]. Moreover, the inhibition of miR-33 in African green monkeys increased hepatic abca1 expression and the HDL-C plasma levels and decreased plasma very low density lipoprotein (VLDL) levels [19].

Aberrant lipid homeostasis, which is implicated in conditions such as dyslipidemia, is associated with obesity and metabolic syndrome. These pathologies represent an important health problem for developed and developing countries [20], [21]. The use of dietary compounds that reduce the health complications related to these pathologies is appearing as a new strategy. It has been reported that polyunsaturated fatty acids (PUFAs) and polyphenols have the capacity to affect cardiovascular diseases, improving lipid homeostasis [22], [23]. Specifically, a grape seed proanthocyanidin extract (GSPE) was shown to reduce plasma TG, apo B and low density lipoprotein-cholesterol (LDL-C) and increase the percentage of HDL-C in healthy rats given an acute oral dose of GSPE [24] and in dyslipidemic rats given chronic administration [25]. Additionally, the intake of ω-3 PUFAs is helpful in the prevention of cardiovascular disease [26]. Hence, the beneficial effects of ω-3 PUFAs on the treatment of hyperlipidemia have been extensively studied in both humans [27] and animals [28], [29], showing a potent hypolipidemic effect. Regarding the mechanism of action of both proanthocyanidins and ω-3 PUFAs, the evidence has recently shown that these dietary compounds can modulate miRNAs expression. Specifically, different FAs have been shown to exert certain biological effects through the direct modulation of miRNAs expression [30]–[32]. Moreover, there are several examples in the literature that demonstrate the capacity of polyphenols to modulate miRNAs [33]; for example, we recently reported the ability of grape seed proanthocyanidins to downregulate liver miR-33a and miR-122 in rats [34].

Regardless of the well-defined roles of miR-33 and miR-122 in controlling lipid metabolism in genetic models, the association of these miRNAs with lipemia in pathophysiological conditions is not well understood. Therefore, the aim of this study was to evaluate whether the levels of miR-122 and miR-33 in the liver correlate well with lipemia that has been nutritionally (non-genetically) induced in different rat models. For this purpose, we analyzed liver miRNA-33a and miR-122 levels in dyslipidemic cafeteria diet- (CD) fed rats and in rats fed a CD supplemented with proanthocyanidins and/or ω-3 PUFAs, two dietary components that are known to counteract dyslipidemia. While determining miRNAs levels in tissues remains important, peripheral blood mononuclear cells (PBMCs) are becoming a non-invasive way to study gene and miRNAs expression because they can be easily collected from the blood and reflect, as biomarkers, the pathological and physiological state of the organism [35], [36]. Therefore, we also evaluated whether miR-33a is expressed in PBMCs and if the changes in liver miR-33a and abca1 expression are reflected in these cells.

## Materials and Methods

### Grape seed proanthocyanidins extract

The GSPE was kindly provided by Les Dérives Résiniques et Terpéniques (Dax, France). The following GSPE composition used in this study has been previously analyzed [37]: catechin (58 µmol/g), epicatechin (52 µmol/g), epigallocatechin (5.50 µmol/g), epicatechin gallate (89 µmol/g), epigallocatechin gallate (1.40 µmol/g), dimeric procyanidins (250 µmol/g), trimeric procyanidins (1568 µmol/g), tetrameric procyanidins (8.8 µmol/g), pentameric procyanidins (0.73 µmol/g) and hexameric procyanidins (0.38 µmol/g).

### Oil rich in docosahexaenoic acid

The DHA-OR was kindly provided by MarteK DHA™ –S. The nutritional oil used in the experiment is derived from the marine alga, *Schizochytrium* sp., a rich source of ω-3 DHA with sunflower lecithin and rosemary extract (flavoring). The fatty acid profile of DHA-OR was 5.6% of 14∶0, 16.1% of 16∶0, 0.9% of 18∶0, 15% of 18:1n-9, 1.4% of 18:2n-6, 0.5% of 20:4n-6 (ARA), 1.1% of 20:5n-3 (EPA), 16.2% of 22:5n-6 (DPA), 38.8% of 22:6n-3 (DHA) and 0.5% of others. The oil also contained tocopherols and ascorbyl palmitate as antioxidants to provide stability.

### Ethic statements

All procedures involving the use and care of animals were reviewed and approved by The Animal Ethics Committee of our university (reference number 4249 by Generalitat de Catalunya).

### Animals

Male Wistar rats weighing 150 g were purchased from Charles River (Barcelona, Spain). The Animal Ethics Committee of our university approved all procedures. The animals were housed in animal quarters at 22°C with a 12 h light/dark cycle (light from 08:00 hours to 20:00 hours) and were fed a standard chow diet (STD) *ad libitum* (Panlab, Barcelona, Spain). After one week, the rats were divided into 5 groups (n = 7): the STD control group, where rats were fed STD *ad libitum*, and 4 other groups that were fed a STD plus a CD as a high fat model which had 23.4% lipids (0.05% cholesterol), 35.2% carbohydrates and 11.7% protein. The CD consisted of the following foods: cookies with foie-gras and cheese triangles, bacon, biscuits, carrots and sugary milk. After 10 weeks, rats feeding on the CD were trained to lick arabic gum (1 mL) (G9752, Sigma-Aldrich, Madrid, Spain), which was used as the vehicle; were divided into 4 groups and were fed a CD plus treatments for 3 more weeks. The first group was supplemented every day with 25 mg GSPE/kg bw dissolved in arabic gum (CD-GSPE group). The second group was supplemented every day with a dose of DHA-OR equivalent to 515 mg ω3-PUFAs/kg bw dissolved in arabic gum (CD-DHA-OR group). The third group was supplemented every day with 25 mg GSPE/kg bw plus a dose of DHA-OR equivalent to 515 mg ω-3 PUFAs/kg bw dissolved in arabic gum (CD-GSPE+ DHA-OR group). The fourth group received the same volume of arabic gum (CD control group). All treatments were administered at the same time point (7 p.m.). After 3 weeks of treatments, rats were killed at 9 a.m. by anesthetizing them with 50 mg/kg bw of sodium pentobarbital (0804118, Fagron Iberica, Terrasa, Spain), and they were sacrificed by bleeding. Blood was collected using heparin (Deltalab, Barcelona, Spain) as an anticoagulant. Plasma was obtained by centrifugation (1500× *g*, 15 min, 4°C) and stored at −80°C until analysis. The livers were excised, frozen immediately in liquid nitrogen and stored at −80°C until RNA and lipids could be extracted.

### RNA extraction

Total RNA containing small RNA species was extracted from frozen liver and PBMCs using a mi/mRNA extraction kit (miRNA kit, E.Z.N.A., Omega Bio-tek, Norcros, U.S.A.) according to the manufacturer's protocol. To isolate both total RNA and miRNA, 1.5 volumes of absolute ethanol was added instead of the recommended 0.33 volumes in step 5. The washing step was performed according to the isolation of large RNAs. The quantity of the purified RNA was determined using a NanoDrop 1000 Spectrophotometer (Thermo Scientific).

### microRNA quantification by real-time qRT-PCR

To analyze the expression of each miRNA, reverse transcription was performed using the TaqMan MicroRNA Reverse Transcription Kit (Applied Biosystems, Madrid, Spain) and the miRNA-specific reverse-transcription primers provided with the TaqMan® MicroRNA Assay (Applied Biosystems, Madrid, Spain). For the reverse transcription, a My Gene L Series Peltier Thermal Cycler (Long Gene) was used. The final total RNA concentration used was 2.5 ng/µL. The reaction was performed at 16°C for 30 min, 42°C for 30 min and 85°C for 5 min. We used 1.33 µL obtained cDNAs in a subsequent quantitative qRT-PCR amplification using the TaqMan Universal PCR master mix (Applied Biosystems, Madrid, Spain) and the associated specific probe provided in the TaqMan® MicroRNA Assay Kit (Applied Biosystems). Specific Taqman probes were used for each gene: microRNA-122a (miR-122a: hsa-mir-122a), 5′UGGAGUGUGACAAUGGUGUUUG-3′ and microRNA-33 (miR-33: hsa-mir-33), 5′- GUGCAUUGUAGUUGCAUUG-3′. The results were normalized to the expression of the U6 small nuclear RNA (U6 snRNA), which was used as an endogenous control. Amplification was performed using the ABI Prism 7300 SDS Real-Time PCR system (Applied Biosystems, Madrid, Spain) at 95°C for 10 min, followed by 40 cycles of 95°C for 15 s and 60°C for 1 min. The fold change in the miRNA level was calculated by the log 2 scale according to the equation 2−ΔΔCt, where ΔCt = Ct miRNA-Ct U6 and ΔΔCt = ΔCt treated samples- ΔCt untreated controls.

### mRNA quantification by real-time qRT-PCR

mRNA levels were evaluated by reverse transcription performed using the High Capacity cDNA Reverse Transcription Kit (Applied Biosystems, Madrid, Spain). For the reverse transcription, a My Gene L Series Peltier Thermal Cycler (Long Gene) was used. The final total RNA concentration used was 25 ng/µL. The reaction was performed at 25°C for 10 min, 37°C for 120 min and 85°C for 5 sec. We used 5 µL obtained cDNA solution for subsequent quantitative RT-PCR amplifications using the TaqMan Universal PCR master mix (Applied Biosystems, Madrid, Spain). Specific Taqman probes were used for each gene: Abca1 (Rn00710172_m1), Fasn (Rn00569117_m1), Cpt1a (Rn00580702_m1), Pparβ/δ (Rn00565707_m1) and Srebp2 (Rn01502638_m1). The results were normalized to cyclophilin (PPIA: Rn00690933_m1), which was used as an endogenous control. Amplification was performed using the ABI Prism 7300 SDS Real-Time PCR system (Applied Biosystems, Madrid, Spain) with a protocol of 50°C for 2 min, 95°C for 10 min and 40 cycles of 95°C for 15 s and 60°C for 1 min. The fold change in the mRNA levels was calculated by the log 2 scale using the equation 2−ΔΔCt, where ΔCt = Ct miRNA-Ct U6 and ΔΔCt = ΔCt treated samples- ΔCt untreated controls.

### Plasma and liver lipid analysis

Plasma total cholesterol and TG were measured with an enzymatic colorimetric kit (QCA, Barcelona, Spain). Liver (0.05 g) was used to extract total lipids using gravimetric analysis. Lipids were dissolved in 1 mL LPL buffer containing: PIPES disodium salt (P3768, Sigma-Aldrich, Madrid, Spain), MgCl_2_x6H_2_O (M9272, Sigma-Aldrich, Madrid, Spain), albumin free FAs (A8806, Sigma-Aldrich, Madrid, Spain) and 0.1% SDS (L3771, Sigma-Aldrich, Madrid, Spain). The TG and cholesterol concentrations in the dissolved extract were measured using QCA enzymatic colorimetric kits (QCA, Barcelona, Spain) following the manufacturer's protocols.

### Isolation of primary rat monocyte-derived macrophages

Rat PBMCs (peripheral blood mononuclear cells) were isolated from total blood by density gradient centrifugation using Histopaque®-1077 (10771, Sigma-Aldrich, Madrid, Spain) according to the manufacturer's protocol. Histopaque®-1077 is a solution of polysucrose (5.7 g/dL) and sodium diatrizoate (9 g/dL) adjusted to a density of 1.077+/−0.001 g/mL. The isolated PBMCs were dissolved in a lysis buffer to obtain total RNA.

### Statistical analyses

The results are reported as the mean ± S.E.M. of seven animals for the group. Groups were compared with one-way ANOVA (p≤0.05) using SPSS software.

## Results

### GSPE and oil-rich in docosahexaenoic acid treatments improve the atherogenic lipid profile induced by a CD

Feeding rats a CD for 13 weeks significantly increased their body weight (bw) by 20% (443 to 531 g bw) and increased plasma TG, total cholesterol (TC) and LDL-C levels compared with those of rats fed a standard chow diet (STD), thus worsening the atherosclerosis risk ratio, HDL-C/LDL-C (table 1). Once obesity and dyslipidemia were induced in rats (i.e., after 10 weeks of CD), rats were fed GSPE and/or oil-rich in docosahexaenoic acid (DHA-OR) for 3 more weeks. The results showed that GSPE administration for 3 weeks returned plasma TG and LDL-C levels to normal values, whereas it induced only a slight reduction of TC. Moreover, rats treated with GSPE showed a ratio of HDL-C/LDL-C that was even higher than that of rats fed a STD. On the other hand, 3 weeks of DHA-OR treatment did not significantly reduce plasma TG levels, but rather normalized the levels of plasma TC and LDL-C and the HDL-C/LDL-C ratio. When GSPE and DHA-OR were simultaneously administered for 3 weeks, this corrected all the dyslipidemic effects induced by the CD because plasma TG, LDL-C, TC and the ratio of HDL-C/LDL-C were normalized.

**Table 1:** Plasma lipids levels of rats fed with STD or CD with or without a GSPE and/or DHA-OR in chronic treatments.

Plasma parameters	STD	CD control	CD+GSPE	CD+DHA-OR	CD+GSPE+DHA-OR
**TG (mg/dL)**	64.13±6.45a	104.37±12.75b	66.32±6.80a	84.07±10.55ab	66.77±6.63a
**TC (mg/dL)**	50.65±2.76a	72.42±1.91b	67.24±1.02b	51.49±2.34a	54.77±3.99a
**HDL-C (mg/dL)**	33.24±2.22	40.75±0.55	49.47±1.89	32.87±1.44	34.78±2.23
**LDL-C (mg/dL)**	5.52±0.39a	13.14±1.43b	5.54±1.10a	6.04±1.01a	6.93±1.53a
**HDL-C/LDL-C**	6.24±0.66	3.92±0.56	8.03±1.40	6.66±1.26	3.99±0.84
**TC/HDL-C**	1.53±0.04	1.68±0.11	1.57±0.15	1.52±0.04	1.60±0.13

*Abbreviations: GSPE, grape seed proanthocyanidin extracts; CD, cafeteria-diet; STD, standard chow diet; DHA-OR, oil-rich in d^ocosahexaenoic acid^; TG, triacylglyceride; TC, total cholesterol. Rats were fed with STD (STD group) or with STD plus CD for 10 weeks. After 10 weeks, rats fed a STD plus a CD were orally treated with 25 mg GSPE/kg bw (CD+GSPE), 515 mg PUFAs/kg bw (CD+DHA-OR), 25 mg GSPE and 515 mg ω-3 PUFAs/kg bw (CD+GSPE+DHA-OR group) or vehicle (CD control group) for 3 weeks simultaneously with the CD. Each value is the mean ± s.e.m. of seven rats. Letters denotes a significant difference between groups (p<0.05; One-way ANOVA)*.

Liver lipid parameters were analyzed by gravimetric analysis (table 2). The CD control group showed an increase in the total lipid content, increasing TG and TC levels. However, no difference was observed in the liver weight compared to livers from rats that were fed a STD. Nevertheless, after the GSPE treatment, the total liver lipids were reduced with a decrease in TG that was comparable to the levels in the CD control. After the DHA-OR treatment, there was no effect on the liver lipid content. Moreover, when GSPE and DHA-OR were simultaneously administered, TC levels were decreased. Therefore, our results demonstrated that GSPE and DHA-OR treatments improved the atherogenic lipid profile caused by a CD.

**Table 2:** Liver weight and liver lipids in rats fed a STD or CD with or without a GSPE and/or DHA-OR in chronic treatments.

Liver parameters	STD	CD control	CD+GSPE	CD+DHA-OR	CD+GSPE+DHA-OR
**Liver weight (%)**	3.01±0.01a	3.06±0.06ab	2.94±0.05a	3.23±0.04b	3.19±0.04b
**Total lipids (g/100 g liver)**	2.73±0.25a	6.94±0.59b	3.79±0.09c	7.00±0.88b	6.50±0.28b
**TG (g/100 g liver)**	0.44±0.03a	1.59±0.12b	0.85±0.08c	2.03±0.40bc	1.60±0.27bc
**TC (g/100 g liver)**	0,15±0.02a	0.69±0.13bc	0.31±0.02b	0.65±0.10bc	0.66±0.05c

*Abbreviations: GSPE, grape seed proanthocyanidin extracts; CD, cafeteria-diet; STD, standard chow diet; DHA-OR, oil-rich in d^ocosahexaenoic acid^; TG, triacylglyceride; TC, total cholesterol. Experimental details and symbols as in* *Table 2*.

### CD increased miR-33a and miR-122 levels in the liver and modulated the expression of their target genes

**Figure 1:**
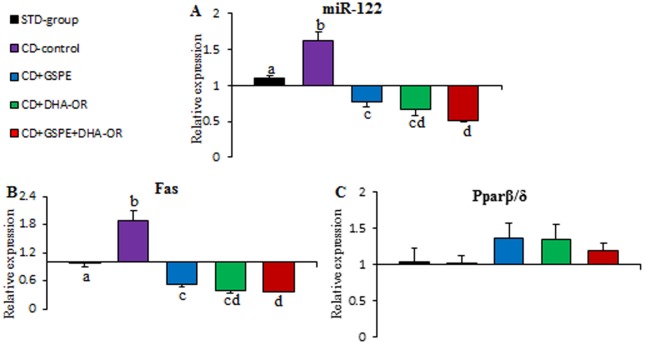
Liver miR-122 and their target mRNA levels. Rats were fed a STD (STD group) or a STD plus CD for 10 weeks. After 10 weeks, rats fed a STD plus CD were orally treated with 25 mg GSPE/kg bw (CD-GSPE group), 515 mg ω-3 PUFAs/kg bw (CD- DHA-OR), 25 mg GSPE and 515 mg ω-3 PUFAs/kg bw (CD-GSPE- DHA-OR group) or vehicle (CD control group) for 3 weeks simultaneously with the CD. Each value is the mean ± s.e.m. of seven rats. Letters denotes a significant difference between groups (p<0.05; One-way ANOVA).

**Figure 2:**
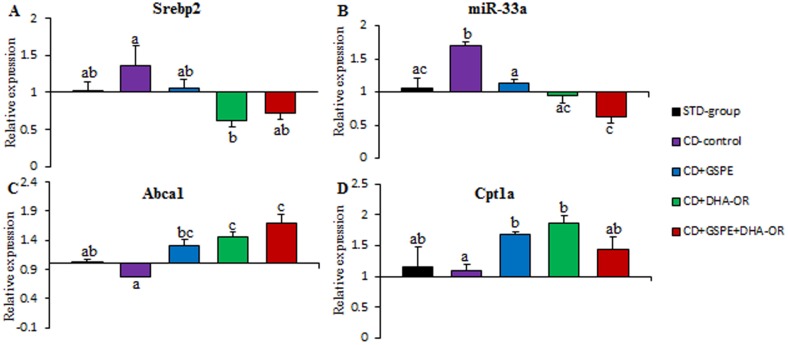
Liver srebp2 and miR-33a and their target mRNA levels. Experimental details and symbols as in Figure 1.

The CD was able to alter the levels of lipid metabolism-related miRNAs (Figures 1 and 2). Specifically, a supplemental CD plus a STD for 13 weeks resulted in an increase in miR-33a levels by 60% and a significant reduction in abca1 target mRNA, with no modification of Cpt1a target mRNA, compared to rats fed only a STD. Similarly, miR-122 expression was upregulated by 47%, and fas target mRNA levels significantly increased, whereas pparβ/δ was no modified, when rats were fed a CD.

### GSPE and DHA-OR reversed the increase in miR-122 and altered their target genes induced by a CD in rat liver

After 3 weeks of GSPE and/or DHA-OR treatments in rats that were fed a CD, the expression of miR-122 in the liver was normalized (Figure 1A). GSPE reduced miR-122 expression by 53%. However, DHA-OR was more effective and reduced miR-122 expression in the liver by 59%. Moreover, when GSPE and DHA-OR were administered simultaneously, the reduction in miRNA expression was greater than when they were administered separately, with reduction in miR-122 expression of 68%. The direct and indirect targets mRNAs levels for miR-122 were also modulated by the three treatments according to their effects on miRNAs levels (Figure 1B and 1C). Therefore, the expression of Fas and Pparβ/δ mRNAs, an indirect and a direct target of miR-122, respectively, were modified according to miR-122 levels. Therefore, fas mRNA was downregulated after treatments, with a greater effect when GSPE and DHA-OR were administered simultaneously. Moreover, Pparβ/δ mRNA levels were increased after treatments, but not significantly.

### GSPE and DHA-OR reversed the increase in miR-33a induced by a CD, whereas only DHA-OR repressed significantly srebp2 in liver

After 3 weeks of GSPE and/or DHA-OR treatments in rats that were fed a CD, the expression of miR-33a in the liver was normalized (Figure 2B). However, the srebp2 mRNA, which encodes miR-33a and are known to be cotranscribed, was no significantly modified by GSPE. Moreover, similar to miR-122, DHA-OR was more effective reducing miR-33a expression in the liver than GSPE. Moreover, DHA-OR also repressed significantly srebp2 comparing with CD. Furthermore, when GSPE and DHA-OR were administered simultaneously, the reductions of miR-33a and srebp2 expressions were greater than when they were administered separately. The targets mRNAs levels for miR-33a were also modulated by the three treatments according to their effects on miRNA level (Figures 2C, 2D). Therefore, Abca1 and Cpt1a mRNAs were upregulated after GSPE and DHA-OR treatments, and when GSPE and DHA-OR were administered simultaneously, the increase in Abca1 mRNA was greater than when they were administered separately.

### PBMCs reflected the liver miR-33a and abca1 mRNA levels

**Figure 3:**
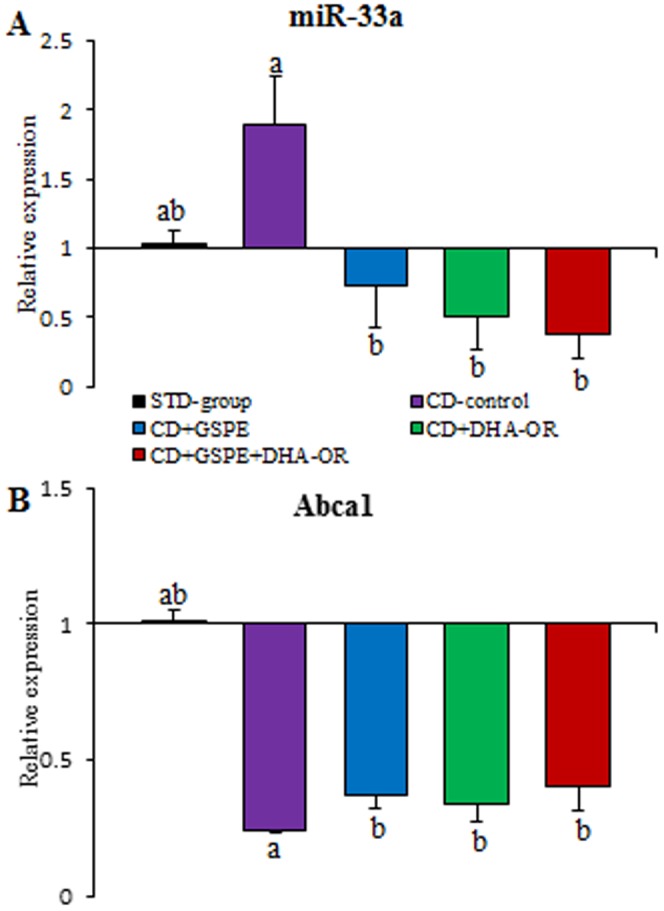
miR-33a and abca1 mRNA levels in peripheral blood mononuclear cells (PBMCs). Experimental details and symbols as in Figure 1.

Figure 3 shows the levels of miR-33a and abca1 in the PBMCs of rats that were fed a CD and rats that were fed a CD supplemented with GSPE and/or DHA-OR. The abca1 mRNA expression in PBMCs reflected that of the liver profile: abca1 mRNA was downregulated by 76% in rats that were fed a CD compared to rats that were fed a STD. As in the liver, GSPE and DHA-OR reversed the effect of the CD, increasing abca1 expression by 54% and 37%, respectively. When the two treatments were administered simultaneously, the effect on abca1 was increased by 66%, as it was in the liver. Moreover, miR-33a expression in the PBMCs of CD-fed rats was upregulated by 81%, which was an even greater increase than in the liver. GSPE and DHA-OR reversed the effect of a CD, reducing miR-33a levels by 62% and 74%, respectively. A greater reduction (80%) was observed when the two treatments were administered simultaneously.

## Discussion

miRNAs have been described as regulators of gene expression, and the deregulation of several miRNAs that are related to chronic diseases has been reported [5]. Specifically, miR-122 and miR-33 play key roles in lipid metabolism. It is well-known that miR-122 is involved in the regulation of several genes in the cholesterol biosynthesis pathway [9] and that it modulates TG metabolism [10]. Additionally, miR-33 plays a crucial role in the regulation of cholesterol metabolism [15], [18], [38], [39]. Although the deregulation of miR-33 and miR-122 has been related to the development of risk factors associated to metabolic diseases, such as obesity and metabolic syndrome, most of the studies on the regulation of lipid metabolism by these two miRNAs have been performed using knockout or antisense models [9]–[12], [17]–[19], [40]. The purpose of this work was to study whether the levels of miR-122 and miR-33a correlate with nutritionally induced lipemia in different rat models; this reflects a more natural physiological state. Therefore, we have analyzed these miRNAs in the livers of dyslipidemic CD-fed rats and in those of rats that were fed a CD supplemented with proanthocyanidins and/or ω-3 PUFAs, which are two dietary components known to counteract dyslipidemia [22], [23].

The CD provides a robust model of human metabolic syndrome compared to traditional lard-based high-fat diets [41]. In this model, animals are allowed free access to a STD and water and are concurrently offered highly palatable, energy dense, unhealthy human foods *ad libitum*. This diet promotes voluntary hyperphagia that results in rapid weight gain and increases fat pad mass and prediabetic parameters, such as glucose and insulin intolerance and dyslipemia [41]. In this experiment, rats fed a CD showed a 20% increase in bw and an atherogenic lipid profile demonstrating a significant increase in TG, TC and LDL-C levels in the plasma. Moreover, rats fed a CD showed increased liver lipids due to the accumulation of both TG and TC.

Interestingly, in association with the atherogenic lipid profile and fatty liver, the levels of miR-122, miR-33a and srebp2, the host gene of miR-33a, were increased in the livers of rats who were fed a CD. In contrast to our results, a downregulation of miR-33 has been described in mice fed a high fat diet (HFD) [38]. However, the effect of a HFD on srebp2 expression is controversial, with studies that demonstrate a downregulation [38] or an overexpression [42] of this gene in mice. Moreover, it should be stated that the severity of hepatic steatosis and inflammation induced by a CD is stronger than that induced by a HFD, with a malignant progression from non-alcoholic fatty liver disease (NAFLD) to non-alcoholic steatohepatitis (NASH) in animals fed a CD [41]. Interestingly, NASH patients have increased expression of srebp2 in liver [43]. Thus, it seems that the type of diet conditions an over- or down-regulation of srebp2 and consequently miR-33 levels.

In relation to miR-122, our results are supported by evidence that plasma miR-122 is increased in patients with hyperlipidemia [44]. However, as with miR-33a, this increase in hepatic miR-122 in rats that were fed a CD has not been observed using other HFD. For instance, mice fed a diet of 58%–60% fat [45], with or without 30% w/v of fructose in the drinking water [46], show a downregulation of miR-122 expression in the liver. Additionally, a reduction in liver miR-122 expression was found in genetically obese ob/ob mice [47]. However, mice that were deficient in LDL receptors and that were fed a diet of 22% fat and 0.32% cholesterol do not show any change in miR-122 expression in the liver [48]. Therefore, like for miR-33a, it seems that diet and genetic modification influence liver miR-122 expression in different ways. Thus, further studies of liver miR-122 and miR-33 expression using different types of diets are warranted.

Direct miR-122 targets that modulate lipid metabolism are essentially unknown and most of the defined target genes of this miRNA, such as Fas, are indirectly modulated. However, recently it has been identified PPARα, β, and γ and the PPARα-coactivator (Smarcd1/Baf60a), as direct targets of miR-122 [49]. For this reason, we have quantified a direct, pparβ/δ, and an indirect, fas, target gene of miR-122. The liver expression of target genes of miR-122 and miR-33a indicate that these rats had increased lipogenesis, decreased fatty acid oxidation and reduced HDL biosynthesis in the liver. Consequently, the increase in miR-122 and miR-33a levels in the liver could explain, to some extent, the dyslipemic effect of a CD.

We used two well-known dietary components that improve lipid metabolism, proanthocyanidins and ω-3 PUFAs (GSPE and DHA-OR, respectively) to determine whether the improvement in the atherogenic profile induced by these compounds is associated with a reduction in liver miR-122 and miR-33a, which were previously increased by the CD. Our results show that a chronic treatment of GSPE in CD-fed rats improved the atherogenic lipid profile caused by the CD, normalizing plasma TG and LDL-C levels and reducing liver TGs and total liver lipids. In contrast, a chronic treatment of DHA-OR in rats that were fed a CD normalized TC and LDL-C plasma levels without an effect on the lipid content of the liver. There are some studies that show that polyphenols and ω-3 PUFAs have a synergistic effect [50]. Thus, we evaluated the hypolipidemic effect of a simultaneous administration of GSPE and DHA-OR. In this case, liver TC and plasma TG, TC and LDL-C levels were decreased to a similar degree to that found when administering the compounds separately, and the effect was not additive or synergistic, but complementary. To confirm the protective effect of GSPE and DHA-OR against cardiovascular diseases, the TC/HDL-C ratio, known as the atherogenic or Castelli index, and the HDL-C/LDL-C ratio were calculated because they are two important indicators of vascular risk with greater predictive value than the isolated parameters [51]. Both treatments resulted in an increase in the HDL-C/LDL-C ratio, reversing the decrease of this index that results from a CD.

Interestingly, these dietary treatments also counteract the overexpression of miR-122, miR-33a that is induced by the CD. Both GSPE and DHA-OR treatments repressed miR-122, miR-33a in the liver, reaching the levels found in rats that were fed a STD. Moreover, when the two treatments were orally administered together, the repression of these miRNAs was greater than when the treatments were administered separately. Previously, we have shown that GSPE represses miR-122 and miR-33a liver expression in rats treated with an acute dose, which also induced postprandial hypolipidemia in normal rats [34]. There is increasing evidence that in the presence of ω-3 PUFAs, different PUFAs, such as DHA, araquidonic acid and gamma linoleic acid, can exert certain biological effects - for example, on the apoptosis pathway - through the direct modulation of miRNAs [32]. However, to our knowledge, there is not any study showing the influence of ω-3 PUFAs on lipid regulator miRNAs such as miR-33 and miR-122. The repression of rat liver miR-122 and miR-33a, induced by GSPE and DHA-OR, was clearly associated with the improvement in the plasma lipid profile that was induced by these two treatments. Therefore, as both treatments repress these miRNAs in the liver and normalize plasma lipids, it could be suggested that the modulation of miR-122 and miR-33a could be one of the molecular mechanisms used by proanthocyanidins and ω-3 PUFAs to improve the plasmatic atherogenic profile that was induced by a CD.

The inhibition of miR-122 by antisense oligonucleotides reduces the biosynthesis of cholesterol and FAs and increases the oxidation of FAs in the livers of normal mice, and it reduces both hepatic cholesterol accumulation and the development of a fatty liver during the development of diet-induced obesity in mice [10]. But, despite the fact that both treatments repressed the expression of miR-33a and miR-122 in the liver to a similar degree, only the GSPE treatment was effective in reducing the liver lipid content. However, it has been reported that rats fed a high fat diet combined with ω-3 PUFA supplementation were protected against steatosis [52]. Nevertheless, our results do not show any modification of lipids in the livers of rats that were fed a CD and treated with DHA-OR. Thus, it should be stressed that the reduction in hepatic levels of miR-122 and miR-33a was associated with the reduction of lipids in the plasma but not in the liver.

In order to elucidate the mechanism by which GSPE and DHA-OR treatments can repress miR-33a in the liver, the expression of the host gene of miR-33, srebp2, was also evaluated. Analyzing the percentage of decrease of srebp2 and miR-33 levels it seems that the mechanisms repressing miR-33 by DHA-OR or GSPE are not exactly the same for each treatment. The percentage of repression of srebp2 (55%) accounted for the percentage of repression of miR-33a (45%) with DHA-OR supplementation, hence indicating that DHA-OR repress miR-33a by repression of its host-gene. On the contrary, the percentage of repression of miR-33a (33%) was higher than the percentage of repression of srebp2 (20%) with GSPE supplementation, suggesting that the repression of srebp2 only partially contributed to miR-33a downregulation.

The effects of GSPE and DHA-OR on the levels of fas, pparβ/δ, cpt1a and abca1 mRNA in the liver were consistent with the repression of miR-33a and miR-122 that was induced by these treatments. The abca1 and cpt1a mRNA, which are repressed by miR-33a [53], were overexpressed with a synergistic effect in abca1 mRNA levels after GSPE and DHA-OR treatments, increasing abca1 expression when the two treatments were orally administered simultaneously. This synergistic effect on abca1 overexpression agrees with the increased ability of the combination of GSPE and DHA-OR to repress miR-33a expression over that of each treatment alone. The fas mRNA was repressed by GSPE and DHA-OR is consistent with the observation that mRNAs involved in lipogenesis tend to be downregulated when miR-122 is inhibited [10], [54], whereas pparβ/δ mRNA levels were no modified significantly. Once again, there was a powerful effect on fas repression when the two treatments were orally administered simultaneously, a situation that was more effective in repressing miR-122 in the liver than was administrating the compounds separately. Therefore, in the liver, there was a direct association between miR-33a and miR-122 levels and the expression of their target genes: abca1 and fas, respectively.

PBMCs are being used in gene expression studies because they can be easily collected from blood and can reflect the pathological and physiological state of an organism in a non-invasive way [35], [36]. Therefore, miR-33a and its target gene, abca1, were analyzed in PBMCs from rats, demonstrating the expression of miR-33a in PBMCs. Furthermore, in all studied groups of rats, miR-33a and abca1 expression in PBMCs reproduced the changes in expression in the liver. Therefore, PBMCs reflected the modifications in the liver that were induced by diet and treatments. Notably, the response of PBMCs to these nutritional interventions was magnified for miR-33a; the degree to which miR-33a was downregulated in PBMCs was always greater than in the liver. This increased response was also observed for abca1 expression in rats that were fed a CD. However, abca1 mRNA levels in PBMCs from rats in the treated groups were similar to or less than those observed in the liver. Our data give evidence that PBMCs can enhance both liver miR-33a and abca1 expression. Therefore, analyzing miR-33a in PBMCs could provide information about the state of miR-33a expression in the liver in a non-invasive way and therefore give information about the physiological state of the patient.

More studies are necessary to elucidate the exact mechanism by which GSPE and DHA-OR repress miR-122 and miR-33a. miR-122 is the most abundant miRNA in the liver and is expressed as a unique miRNA within a single transcript, *hcr* [54], whereas both miR-33a and b are intronic miRNAs located within Srebf2 and Srebf1, respectively [53]. These loci encode the membrane-bound transcription factors SREBP1 and SREBP2, which activate FA synthesis and cholesterol synthesis and uptake [55]. Therefore, it could be possible that proanthocyanidins and ω-3 PUFAs modulate miR-33a by controlling host gene expression.

In conclusion, the levels of miR-122 and miR-33a in the liver correlate with a state of lipemia in physiological rat models that were nutritionally induced by a cafeteria diet or a cafeteria diet plus hypolipidemic dietary compounds (i.e., GSPE and/or ω-3 PUFAs). Furthermore, we demonstrated that miR-33a is expressed in PBMCs and correlates well with miR-33a liver expression. Hence, the PBMCs could be used as a non-invasive diagnostic or therapeutic biomarker for the levels of liver miR-33.
